# Effect of Combined Regulatory Behavior Index (CRBI) on regulatory fatigue and behavioral adaptability (classical and reformulated) in the university context

**DOI:** 10.3389/fpsyg.2025.1533725

**Published:** 2025-08-04

**Authors:** Jesús de la Fuente, Ena Ubani, Evangelia Karagiannopoulou, Douglas F. Kauffman

**Affiliations:** ^1^Department of Psychology, School of Education and Psychology, University of Navarra, Pamplona, Spain; ^2^Department of Psychology, School of Social Sciences, University of Ioannina, Ioannia, Greece; ^3^Department of Clinical Educational and Health Psychology, University College London, London, United Kingdom; ^4^Independent Research, Greater Boston Area, MA, United States

**Keywords:** combined regulatory behavior index (CRBI), regulatory fatigue, classical adaptability, reformulated adaptability, college students

## Abstract

**Introduction:**

Regulatory fatigue is a potential explanatory mechanism for dysfunctional regulatory behavior, which would lead to poorly adaptive behavior. Based on this premise, it was hypothesized that it would have a significant mediating effect between the combined regulation level (average internal-external regulation) and the students' adaptability.

**Method:**

A total of 365 undergraduates completed, at three points in time, two standardized inventories during a 4-month period. Under an ex post-facto design, linear, inferential and mediational analyses were carried out to verify these effects.

**Results:**

The results showed that the combined regulation level was a significant predictor of fatigue and adaptability, as well as fatigue with respect to adaptability. This effect was corroborated at the inferential level. The most significant model showed the mediational value of fatigue between both, the regulation level, and adaptability, with special significance for the reformulated adaptability.

**Discussion:**

The implications and limitations of the work are discussed. The relevant role of the combined level of regulation (personal and contextual) is noted, and above all, the relevance of regulatory fatigue as a mechanism that encourages the transition from regulated behavior to non-regulated behavior (deregulated) and, finally, to dysregulated behavior.

## Introduction

The problem of stress and academic burnout has been a topic of research interest due to its impact on academic achievement and adaptability among university students (Collie et al., 2016; Martin, 2014). Earlier works identified that regulatory fatigue and sustained academic stress give rise to exhaustion or a state of ego depletion in high-performance university settings (Hagger and Chatzisarantis, 2016; Hagger et al., 2010). Research in the field has also shown that adaptability is significant in university contexts (Martin et al., 2016). However, although adaptability is a known phenomenon, there is no single detailed understanding of the relation between pre-existing levels of personal and contextual regulation and adaptability. There is an imperative need to develop such an understanding, particularly for the most vulnerable students with greater risk factors or who need protection (Li and Song, 2024). For that reason, the aim of this research is to contribute to clarifying those relationships in university students, on the assumption that regulatory fatigue may be an explanatory variable that mediates to some degree changes in behavioral regulation that in turn affect adaptability in students (Schmeichel et al., 2010).

### The conceptual utility model for the management of stress and mental wellbeing as starting point

The *Conceptual Utility Model for the Management of Stress and Psychological Wellbeing*, Spanish acronym CUMSPW^®^ (de la Fuente and Martínez-Vicente, 2023b,c) was created in order to assess and treat stress and mental health in different areas of the practice of psychology, including university settings (see Annex 1). In structural terms, this conceptual utility model is based on the 3Ps Biggs model (Presage-Process-Product; Biggs, 2001). This model was initially conceived to analyze the factors implicit in the processes of learning and university performance, but it can be applied to the protective and risk factors of academic stress:

1) *Presage variables*. Under the model, presage or predictive variables can be both personal and contextual (de la Fuente and Martínez-Vicente, 2023a). The *Theory of Self-vs. Externally-Regulated Behavior, SR-ER*^®^ (de la Fuente-Arias, 2017; de la Fuente, 2021a; de la Fuente et al., 2022a,b; de la Fuente and Kauffman, 2025) allows the assessment of levels of regulation in both the individual and their context. It postulates regulation/non-regulation/dysregulation as discrete levels of both internal and external regulation (de la Fuente and Martínez-Vicente, 2023a).
2) *Process Variables (mediator or buffer variables)*. The model identifies the different types of learning that the individual needs to acquire in order to be competent in the management of stress and mental health. That allows an analysis of variables or subcompetencies of different kinds: conceptual, procedural, and attitudinal:

1)*Conceptual subcompetencies (knowledge)*: the conceptual variable *character strengths* has been shown to be key to the assessment of individuals' beliefs and ethical principles and their underlying cognitive-motivational strategies in relation to wellbeing and mental health (de la Fuente et al., 2022a,b).
2)*Procedural subcompetencies (know-how)*: procedural variables include higher order skills and meta-skills, which may be meta-cognitive, meta-emotional, meta-behavioral, or meta-motivational. They include resilience, coping strategies, self-regulation, engagement, procrastination, emotional dysregulation and burnout (de la Fuente and Martínez-Vicente, 2023a; Rentzios et al., 2025). *Regulatory fatigue* is a failure of the competency of self-regulation examined in greater depth in this work.
3)*Attitudinal subcompetencies (life skills)*. Attitudinal variables include positivity, positive or negative emotionality or emotional reactivity, action-emotion styles, perfectionism, spirituality and self-compassion, personal character strengths, resilience and behavioral *adaptability*, the focus of this work.

3) *Product variables*. The product variables in the model include experience of academic stress and mental wellbeing (Ryff, 1995; Ryff and Singer, 1996; Stallman, 2010). Those variables allow determination of the levels of stress and mental wellbeing responses (de la Fuente and Martínez-Vicente, 2023a).

### The theory of Self- vs Externally-Regulated Behavior (SR-ER)

The *Theory of Self-vs. Externally-Regulated Behavior* (de la Fuente-Arias, 2017; de la Fuente, 2021a; de la Fuente et al., 2022a,b; de la Fuente and Kauffman, 2025) -as a conceptual model of a *presage* nature- proposes three principles or corollaries:

1) When *regulation* is motivated by internal personal factors of the individual, it is called *personal self-regulation*. Self-regulation can be thought of as the extent to which an individual is proactive in regulating their own behavior (Brown, 1998). When it is environmental stimuli that make the positive or negative directionality of a given behavior more likely, that is known as *external, environmental or hetero-regulation* (de la Fuente-Arias, 2017).
2) Both *self-regulation* and *external regulation* can exist at different levels: Regulatory, Non-Regulatory and Dysregulatory (de la Fuente and Martínez-Vicente, 2023a). The level of *individual self-regulatory competency* can be categorized at the following levels: 3 = high or SR (proactive Self-Regulation); 2 = moderate or NR (cessation or absence of proactive self-regulation); and, 1 = low or DR (dysregulation or dysfunction of regulation) (de la Fuente et al., 2022a,b). On the other hand, a regulatory environment or context can be categorized at the following levels: 3 = high or ER (highly regulatory environment or context); 2 = moderate or ENR (non-regulatory environment or context); and, 1 = low or EDR (dysregulatory or regulatory dysfunctional environment or context) (de la Fuente et al., 2022a,b).
3) The combination of those two sets of factors gives rise to the *Index of Combined Regulatory Behavior* (ICRB), with a range of 1–5, which measures combined internal and external regulation. There are five possible combinations under the Index (de la Fuente et al., 2022a,b).

### The Combined Regulatory Behavior Index (CRBI), as presage variable

Behavioral regulation (cognitive, emotional, and behavioral) is the process by which individuals modulate their cognition and emotions and modify their behavior in order to achieve their goals, adapt to their environment and increase their own wellbeing and that of the community at large (Pérez and Bello, 2017). Behavioral regulation has classically been seen as a personal, meta-behavioral process, but more recent theorisations have conceptualized it as a variable that is dependent on combined personal and environmental regulatory factors.

The concept of *combined behavioral regulation* is based on an interactive person-context behavioral view. Unlike partial views, which focus on one of the two factors, it attempts to probabilize the weight of both to explain self-regulatory variability. Thus, it is assumed that to adequately understand and predict said behavior, it is necessary to assume a combined function of personal and contextual regulatory factors.

The combined regulation level is calculated by summing and dividing by two the internal (3 = high; 2 = medium; 1 = low) and external (3 = high; 2 = medium; 1 = low) regulation levels. The average sum of the three levels produces a range of five score levels (1.00; 1.5; 2.0, 2.5; 3). These averages carry an ordered range from 1 to 5 levels (see Table 1). Level 5 reflects an average score for internal and external regulation of 3; Level 5 denotes *high protection and low risk*; Level 4 is an average score of 2.5 and denotes a moderate-high level of protection; Level 3 is an average score of 2 and denotes moderate or non-regulation and moderate protection; Level 2 is an average score of 1.5 and denotes moderate-low protection; finally, Level 1 is an average score of 1 and denotes *low protection and high risk*. The Index can thus be thought of as a measure of *regulatory status* and associated levels of behavioral protection and risk (see Tables 1, 2).

**Table 1:** Levels of the Combined Regulatory Behavior Index (CRBI)^®^ as postulate by SR vs. ER Theory (de la Fuente-Arias, 2017; de la Fuente, 2021a; de la Fuente et al., 2022a,b).

**Combinations of levels of regulation**		**Mean**	**Rank**	**Regulation tendency**	**Stress protection**	**Stress risk**
**SR level (range)**	**ER level (range)**					
**3** (3.85–5.00) H	3 (2.84–5.00) H	3.0	**5**	**High–high:** *high-regulation*	*High protection*	*Low risk*
**2** (3.10–3.84) M	**3** (2.84–5.00) H	2.5	**4**	**Medium–High**: *regulation*	*M–H protection*	*M–L risk*
**3** (3.85–5.00) H	**2** (2.35–2.83) M	2.5	**4**	**High–medium**: *regulation*	*M–H protection*	*M–L risk*
**2** (3.10–3.84) M	**2** (2.35–2.83) M	2.0	**3**	**Medium:** *non-regulation*	*Medium protection*	*M risk*
**2** (3.10–3.84) M	**1** (1.00–2.34) L	1.5	**2**	**Medium–low**: *dysregulation*	*M–L protection*	*M–H risk*
**1** (1.00–3.09) L	**2** (2.35–2.83) M	1.5	**2**	**Low–medium**: *dysregulation*	*M–L protection*	*M–H risk*
**1** (1.00–3.09) L	**1** (1.00–2.34) L	1.0	**1**	**Low–low:** *high dysregulation*	*Low protection*	*High risk*

Combinations of levels of regulation (1 = L, low; 2 = M, medium; 3 = H, high). Effects analyzed in the investigation. See reports of prior research for analysis of differences (de la Fuente et al., 2019, p. 12; de la Fuente, 2021a, p. 5).

**Table 2:** Calculation of the Combined Regulatory Behavior Index (CRBI).

**Level of personal regulation**	**+**	**Level of contextual regulation/2**	**= Mean level**	** = > Range (regulatory meaning)**
3	+	3	= 3.0	= > 5.0 = **A** (excellent)
3	+	2	= 2.5	= > 4.0 = **B** (fairly good)
2	+	3	= 2.5	= > 4.0 = **B** (fairly GOOD)
2	+	2	= 2.0	= > 3.0 = **C** (could be better)
1	+	2	= 1.5	= > 2.0 = **D** (could be a lot better)
2	+	1	= 1.5	= > 2.0 = **D** (could be a lot better)
1	+	1	= 1.0	= > 1.0 = **E** (could be very much better)

1 = low; 2 = moderate; 3 = high. Mean levels = 1-3. Range: 1–5; Meaning = A–E. Level of personal regulation (1 to 3) + Level of contextual regulation (1 to 3)/2 = Mean Level (1 to 3) => Range (regulatory meaning) (1 = E to 5 = A).

### Behavioral adaptability as an attitudinal variable and the effect of personal regulation

#### Classical behavioral adaptability: high, low

*Behavioral adaptability* has been defined as the capacity to adjust and respond effectively to changes in the environment and to unexpected situations: change, novelty, variability, and uncertainty (Collie and Martin, 2016). It has also been defined as the skill of being able to change or adjust the individual's behavior when faced with different circumstances or people, which roots behavioral adaptability in self-regulation (Martin et al., 2012, 2013a,b). It allows adjustment to the individual's responses to changing externalities and internal processes to meet the demands of a given setting (Folke et al., 2010). The theory of adaptability (Martin, 2017a,b) is a “tripartite” theory that has extended the definition of the term not just to include elements of cognitive and behavioral regulation but also emotional regulation in response to change, variability and uncertainty (Martin et al., 2012, 2013a,b, 2015, 2023).

Behavioral adaptability is important in development, because as we all know, over the lifetime of an individual, we will all see a lot of change over the course of our lives (Martin et al., 2013a,b). People face change, novelty and uncertainty at different stages of life, such as starting school, getting on with our peer group, moving home, getting married, etc. (Martin et al., 2013a,b). Those changes disrupt our routines and give rise to new circumstances that the individual has not previously encountered (Martin et al., 2013a,b). How we adapt to those changes will in part determine our wellbeing (Holliman et al., 2018; Martin et al., 2013a,b).

Given its characteristics, the conceptual model of stress management and wellbeing has treated behavioral adaptability as a variable at the level of *attitudinal subcompetency* because it comprises elements of that subcompetency: cognitive, emotional and behavioral adaptation or flexibility. In parallel, behavioral adaptability has been shown to be strongly associated with *self-regulation*, as a procedural subcompetency or meta-skill essential to the management of stress and wellbeing (de la Fuente, 2021a) and *academic buoyancy* (Bostwick et al., 2022; Putwain et al., 2024).

#### A reformulation of behavioral adaptability: adaptation, non-adaptation and dys-adaptation

Under *SR vs. ER Behavior Theory* (de la Fuente-Arias, 2017; de la Fuente et al., 2022a,b, 2024), adaptive behavior can be placed on a continuum at one of three levels: (1) adaptive, (2) non-adaptive and (3) maladaptive in function of the regulatory effort exerted: (1) *adaptive* behavior arises when there are behaviors of change or adjustment as a result of a good level of regulatory effort; (2) *non-adaptive* behavior (or de-adaptative) arises when regulatory effort starts to decrease as a result of accumulated fatigue and adaptive effort starts to decline; (3) *maladaptive* behavior arises when an individual reaches a state of regulatory exhaustion and starts to show maladaptive behavior excesses and deficits (de la Fuente, 2021a).

The *revised adaptability model* assumes in essence the same principle as the concept of self-regulation as behavior that can change in function of effort and the directionality of regulatory behavior from (1) a state of *self-regulation*, or adaptation, toward a (2) state of *non-regulation* (when the individual ceases to exert a regulatory effort), to end at (3) a state of *dysregulation* (involving behavioral excesses and deficits). Thus, it is assumed that behavioral adaptability can be at one of three levels: (1) behavioral adaptability (adaptation through adaptive effort); (2) behavioral non-adaptability (cessation of adaptive effort); (3) behavioral maladapt ability (dysfunctional or undesired adaptation, as a result of adaptive rigidity or changeability—behavioral excesses or deficits) (de la Fuente, 2021a). To measure those levels, the *Revised Adaptability Scale* (de la Fuente, 2021a) used in this article (see Complementary material) was created.

#### Regulatory fatigue as an explanatory mechanism mediating between behavioral regulation and adaptability

Self-regulation, in the sense of the meta-skill of being able to control of the individual's dominant impulses or responses and hence their thoughts and emotions, is determinative of a wide range of socially significant behaviors (Baumeister and Newman, 1994). However, behavioral adaptability is a capacity or behavior of limited capacity. According to the *strength model* (SM), a very influential theory of self-regulation, all actions directed toward self-regulation draw on a limited source of energy (Baumeister and Vohs, 2007). Self-regulatory effort depletes that strength and leads to temporary regulatory fatigue which in turn leads to failures of regulatory behavior (Muraven et al., 1998). Since any effort to internally or externally control mental or physical activity is part of self-regulation, the self-regulatory capacity of individuals varies (Carver and Scheier, 2001). Research has shown that self-regulatory efforts, such as controlling thoughts, impulses and emotions, are associated with lesser persistence in subsequent tasks, an effect known as *ego depletion* (Muraven et al., 1998) or *self-regulatory fatigue* (SRF). The underlying SM hypothesis is that self-regulatory efforts draw on a limited resource and can be exhausted or depleted, in a similar way to a muscle (Muraven et al., 1998). Examining the effect of burnout on task performance and the results in 82 separate studies, a recent meta-analysis found support for the effect of exhaustion and the hypothesized strength model, but suggested that alternatives such as lack of motivation, fatigue and negative affect may play a role (Hagger et al., 2010). In fact, low self-regulatory effect in patients was associated with low persistence similar to that shown by patients and controls who make high self-regulatory efforts, which suggests that patients with chronic multi-symptom illness may in fact have chronic self-regulatory fatigue (Nes et al., 2011).

A recent analysis examining the influence of fatigue on self-control found that fatigue only consistently adversely impacts self-control in specific circumstances. The study argues that fatigue has an indirect rather a direct impact on the performance of self-control, which determines how intensely an individual resists the impulse to act. The paper also argues that the effect of fatigue on intensity of restraint is multifaceted, depending on the level of fatigue, the perceived difficulty of the challenge faced and the importance of resisting an impulse. According to the paper's analysis, *regulatory fatigue* may lead to three distinct outcomes: (1) First, it may lead individuals to more strongly resist (self-regulatory behavior); (2) second, it may lead individuals to disconnect and give in to an impulse (non-regulatory behavior); (3) it may confirm an individual's existing inclination not to resist, doing the opposite of what they want, think or feel (dysregulatory behavior). In the round, the paper allows us to postulate three distinct levels of *adaptive behavior*: adaptive, non-adaptive and maladaptive (de la Fuente, 2021a).

*Regulatory fatigue* is notably observed in cases of ADHD. The evidence suggests that individuals with ADHD experience greater irritation, fatigue, constant need for novelty or stimulation, and variability in task performance or persistence (de la Fuente et al., 2022a,b; Rojas, 2011). Regulatory fatigue has also been associated with personality traits, whereby individuals who have conscientiousness and optimism show lesser regulatory fatigue (Inzlicht and Schmeichel, 2012; Nes et al., 2011). That gradated relationship with self-regulation has recently been demonstrated (de la Fuente et al., 2024).

### Aims and hypotheses

#### Levels of combined (internal and external) regulatory fatigue and behavioral adaptability

As noted, the relationship between combined regulation, regulatory fatigue and levels of adaptability is plausible, albeit it has not been definitively determined. SR-ER theory predicts that an individual's level of combined regulation will determine their level of personal adaptability such that a higher level of internal-external regulation will lead to a higher level of personal adaptability, and vice versa. In this hypothesized relationship, regulatory fatigue is also an explanatory mediating mechanism such that a high level of personal and contextual regulation entails lesser regulatory effort and regulatory fatigue, and conversely lesser regulatory fatigue favors greater behavioral adaptability. The principle applies the other way round, such that the worse an individual's level of combined regulation the greater their level of regulatory effort and regulatory fatigue and, in turn, the lower their level of behavioral adaptability. Thus, regulatory fatigue is an explanatory mediating factor for behavioral adaptability. However, that remains to be shown, which is the purpose of this work.

#### General objectives

This work seeks to examine the relationship between three variables: (1) combined internal and external regulation (presage variable); (2) regulatory fatigue (procedural process variable) and, (3) behavioral adaptability (attitudinal process variable) in order to determine their associations and predictive power. And in order to determine the implications for assessment and intervention in psychiatric and psychological support to university students of the variables examined.

#### Hypotheses

*Association and linear prediction relationships*

1) The level of combined (internal-external) regulation is associated with and significantly negatively predicts regulatory fatigue and significantly positively predicts behavioral adaptability (both as classically formulated and as redefined).
2) Regulatory fatigue is positively associated with and negatively predicts levels of adaptability, most notably for adaptability as redefined.

*Inferential relationships*

3) The score on the index of combined regulation significantly determines differences in the level of regulatory fatigue and behavioral adaptability (classical and redefined).

*Mediating relationships*

4) Regulatory fatigue is a significant behavioral variable that mediates between combined behavioral regulation and behavioral adaptability, with greater significance for adaptability as redefined.

## Methods

### Participants

The sample comprised 354 undergraduate students at publicly funded Spanish universities who voluntarily participated in the study. The sample was a convenience sample made up of students following courses in Psychology, Primary Education and Educational Psychology. The participants were aged 18–25 (mean 20.66; σ 1.86). The majority of participants (71.4%) were women. The inclusion criteria were to be a university student aged 18–25 and to have been invited to participate. The exclusion criterion was a diagnosis of a psychological or psychiatric disorder.

### Instruments

#### Combined internal-external regulation

The Scale of Self-Regulation vs. External Regulation in academic contexts was used (de la Fuente, 2021a). The SRL-ERL Questionnaire (de la Fuente et al., 2022b) was used to measure academic behavioral regulation of individuals and their contexts. The questionnaire consists of three subscales with six items each that assess regulatory, non-regulatory, and dysregulatory aspects of internal regulation, and another three subscales with six items that assess regulatory, non-regulatory, and dysregulatory aspects of external or contextual regulation. The Scale generates individual scores for internal regulation and external regulation from the subscales. Those scores are referred to in what follows as Self vs. External Regulation. The items include: (1) internal regulation: I think consciously about my academic needs; (2) external regulation: the social context that I live in (family, environment, friends) helps me plan my academic behavior by setting goals and objectives; (3) internal non-regulatory: I don't have to make decisions to achieve changes in my academic behaviors; (4) external non-regulatory: the social context that I live in (family, environment, friends) makes me think that you don't need to make specific decisions to make changes in your academic behaviors; (5) internal dysregulatory: there's no point in changing your academic behavior if you have to do less of what you enjoy and find satisfying, and (6) external dysregulatory: the social context that I live in (family, environment, friends) helps me enjoy myself to the fullest, it doesn't encourage me to change my academic behavior, but to do what I feel like, if that makes me happy and live life to the full. The confirmatory factor structure of the questionnaire in this sample was consistent [Chi Square = 1,348.005, df = 583, *p* < 0.001; Ch/df = 2.379; RMSR = 0.035; NFI = 0.967; RFI = 0.954; incremental fit index (IFI) = 0.902; TLI = 0.967; CFI = 0.978; RMSEA = 0.700]. The total reliability value was also acceptable (total alpha = 0.776), as were the subscale consistency values: SRH = 0.847; NRH = 0.779; DRH = 0.769; ERH = 0.900; ENH = 0.761; EDH = 0.828.

#### Self-regulatory fatigue

*Self-Regulatory Fatigue* was measured using the *Self-Regulatory Fatigue Scale*, SRF-18 (Spanish version: de la Fuente, 2021a). The questionnaire asks respondents to indicate the extent to which they agree with a series of statements on a 5-points Likert-type scale (1 “Totally disagree” and 5 “Totally agree”). The instrument has three fatigue subscales: F1 *Behavioral Fatigue* (for example: I feel like hitting, throwing, breaking or squashing things; five items); F2 *Cognitive Fatigue* (for example: I feel full of energy. Inverted item; six items); F3, *Emotional Fatigue* (for example: I easily lose my temper; seven items). The confirmatory structural values for this construct were acceptable [Chi-Square = 427,852, df = (189–57) 132; Chi/df = 32,141; RMR = 0.0357; NFI = 0.956, RFI = 951; IFI: 947; TLI = 0.924; CFI = 0.948; RMSEA = 0.042; Hoelter = 1,234 (*p* < 0.05), 1,457 (*p* < 0.01)], showing three complementary components (behavior, cognitive and emotional fatigue). Reliability was high for the total scale (alpha = 0.783; omega = 0.730), the behavioral fatigue sub-scale (alpha = 0.741; omega = 0.752; item 8 was eliminated because it reduced the reliability of the subscale) and the emotional fatigue subscale (alpha = 0.790; omega = 0.777).

#### Traditional adaptability

Traditional adaptability was measured using *The Adaptability Scale* (Martin et al., 2012; Spanish version: de la Fuente, 2021b). This Scale comprises nine items, of which six items relate to cognitive-behavioral adaptablity and three items to afective adaptability. The scale had adequate reliability (Cronbach's alpha = 0.84; part 1 = 0.861; part 2 = 0.810; Spearman-Brown = 0.819; Guttman's split half reliability = 0.818). The confirmatory structural values for this construct were acceptable [Chi-Square = 435,856, df = (189–57) 132; Chi/df = 32,141; RMR = 0.0257; NFI = 0.943, RFI = 931; IFI: 947; TLI = 0.913; CFI = 0.946; RMSEA = 0.035; Hoelter = 1,346 (*p* < 0.05), 1,256 (*p* < 0.01)], showing three complementary components (behavior, cognitive and emotional fatigue). Reliability was high for the total scale (alpha = 0.792; omega = 0.735), the behavioral fatigue sub-scale (alpha = 0.783; omega = 0.712; item 8 was eliminated because it reduced the reliability of the subscale) and the emotional fatigue subscale (alpha = 0.795; omega = 0.776).

#### Redefined adaptability

Redefined adaptability was measured using the Adaptability Scale Revisited: Adaptability (AD)/Non-adaptability (NA)/Dys-adaptability (DA) (de la Fuente, 2021a), based on Martin et al. (2012). See Supplementary Annex 1. It contains a total of 27 items divided into three groups of nine related to the level of adaptive behavior (adaptive cognitive, emotional and behavioral proactivity), non-adaptive behavior (adaptive inaction or cessation of adaptive cognitive, emotional and behavioral effort), and maladaptive behavior (proactive adaptive cognitive, emotional and behavioral dysfunction). The items include: “I can change how I think about a new situation to help myself to deal with it” (cognitive adjustment); “When an unclear situation arises, I don't reduce my frustration or irritation so as to be able to deal with it better” (emotional non-adjustment); “To help myself to deal with unclear new situations, I have negative feelings and emotions (eg, shame, despair)” (behavioral maladjustment).

The scale's factorial structure had acceptable values. The confirmatory structural values for this construct were acceptable [Chi-Square = 416,845, df = (189–57) 132; Chi/df = 3,457; RMR = 0.0346; NFI = 0.946, RFI = 958; IFI: 949; TLI = 0.923; CFI = 0.9478; RMSEA = 0.052; Hoelter = 1,256 (*p* < 0.05), 1,547 (*p* < 0.01)], showing three complementary components (behavior, cognitive and emotional fatigue). Reliability was high for the total scale (alpha = 0.792; omega = 0.738), the behavioral fatigue sub-scale (alpha = 0.751; omega = 0.750. Cronbach's alpha for the scale was 0.848 (part 1 = 0.751; part 2 = 0.823). Correlation and regression analysis between the classical version and the revised formulation of adaptability showed directionality and consistency (see Tables 3, 4).

**Table 3:** Linear association relationships between classical and revised versions (*n* = 298).

**Revised version**	**Classical version**		
	**F1.ADAPCOGN**	**F2.ADAPEM**	**F3.ADAPTOT**
Adaptation	0.733^**^	0.659^**^	0.762^**^
Non-adaptation	−0.394^**^	−0.350^**^	−0.407^**^
Dys-adaptation	−0.238^**^	−0.300^**^	−0.308^**^

F1: ADAPCOGN: cognitive adaptation; F2: ADAPEM: emotional adaptation; F3: ADAPTOT: total adaptation.

^**^p < 0.01.

**Table 4:** Linear predictive relationships between classical and revised versions (*n* = 298).

**Revised version**	**Beta**	**Traditional version**	***R^2^***	***F***
Adaptation	0.783^***^	ADAPTOTAL	0.600	*F*_(3,267)_ = 135,910^***^
Non-adaptation	0.104			
Dys-adaptation	−0.133^*^			
Adaptation	0.721^***^	ADAPCOGN	0.540	*F*_(3,270)_ = 107,885^***^
Non-adaptation	−0.038			
Dys-adaptation	0.010			
Adaptation	0.700^***^	ADAPEM	0.463	*F*_(3,271)_ = 79,591^***^
Non-adaptation	0.186^**^			
Dys-adaptation	−0.207^**^			

^***^p < 0.001.

^**^p < 0.01.

^*^p < 0.05.

ADAPCOGN, cognitive adaptation; ADAPEM, emotional adaptation; ADAPTOT, total adaptation.

### Procedure

Students participated voluntarily. They first signed an informed consent and then completed the scales anonymously online. The Research Ethics Committee of the University of Navarra (ref. 2018.170) and the University of Almería (ref. UALBIO2024.050) approved the R and D Project. The study was conducted in accordance with the normal ethical principles of the profession of psychology. All databases were anonymised and enjoyed the protections required by laws concerning the protection of personal data. The data server is at (NETERRA DATACENTERS EUROPE1). Mapache Software Europe carries out data processing there subject to all appropriate safeguards. Project IP2 acts as data controller.

### Data analysis

Under an ex post cross-sectional *design* (Ato et al., 2013) three analyses were carried out.

#### Initial analysis

First, data quality was assessed by testing for outliers and missing cases. Univariate outliers were tested for by determining typical scores for each variable. *Z*-scores outside a three-unit range were presumed to be atypical (Tabachnick and Fidell, 2001). The kurtosis of each observed variable determined univariate normality, distribution and asymmetry. Asymmetry values above 3 and kurtosis above 10 prompted data transformation (Kline, 2005). Multicollinearity was tested using bivariate correlation on the basis that correlation of 0.85 or above indicates satisfaction of the assumption. Power of 0.80 was used as the threshold for acceptability.

#### Descriptive analysis

To determine descriptive values central tendency analysis (mean and SD), asymmetry and kurtosis were used. Normal distribution of the sample was first confimed via the Kolmogorov–Smirnoff test.

##### Association and linear prediction (Hypothesis 1)

Parametric analysis was performed via Pearson bivariate correlation and linear regression. Those correlations are better suited to clearly ordinate variables with high values for normality and multivariate kurtosis. Cronbach's alpha was used for total reliability and the reliability of each of the factor structures proposed. SPSS 26 (IBM Corp, 2019) for reliability, and AMOS v. 23 (Arbuckle, 2014) were used for confirmatory factor analyses and SEM.

##### Inferential analysis (Hypothesis 2)

The combined index of behavioral regulation (de la Fuente, 2024a,b) was calculated in the normal way: (1) obtaining the arithmetic mean of scores for regulation on the Regulation–Non-Regulation–Dysregulation heuristic for both internal and external regulation (2) determining high (1), moderate (2) and low (3) levels of personal and external regulation through cluster analysis and upper and lower limits for each level.

MANOVA was subsequently performed to confirm the suitability of the groups [*F*_(24,2,480)_ = 34,772^***^(Pillai) *r*^2^= 0.252; power = 1.0]. Direct and effect values are shown in Table 5.

**Table 5:** Multivariate analysis of the suitability of groups.

**SRL-ERL**	**Levels of Combined Regulatory Behavior Index (CRBI)**						**Partial effect**	**Post**
	**1.0 (*****n*** = **82)**	**1.5 (*****n*** = **116)**	**2.00 (*****n*** = **193)**	**2.5 (*****n*** = **135)**	**3.0 (*****n*** = **101)**	**Mean (*****n*** = **627)**		
SRL	3.59 (0.74)	3.74 (0.63)	3.94 (0.73)	4.11 (0.58)	4.48 (0.92)	3.98 (0.67)	*F*_(4,627)_ = 31,584^**^, *r^2^*= 0.169	3, 2.5, 2.0 > 1.5, 1.0^*^
NRL	3.49 (0.60)	2.80 (0.49)	2.51 (0.49)	2.31 (0.56)	1.73 (0.44)	2.52 (0.51)	*F*_(4,627)_ = 144,518^**^, *r^2^*= 0.482	3, 2.5, 2.0 < 1.5, 1.0^*^
DRL	3.50 (0.61)	2.73 (0.65)	2.54 (0.59)	2.19 (0.63)	1.72 (0.53)	2.49 (0.79)	*F*_(4,627)_ = 110,464^**^, *r^2^* = 0.415	3, 2.5, 2.0 < 1.5, 1.0^*^
ERL	3.51 (0.77)	3.09 (0.95)	3.63 (0.85)	4.01 (0.82)	4.50 (0.54)	3.74 (0.29)	*F*_(4,627)_ = 45,960^**^, *r^2^* = 0.228	3, 2.5, 2.0 > 1.5, 1.0^*^
ENRL	3.61 (0.51)	2.95 (0.71)	2.39 (0.60)	1.89 (0.54)	1.46 (0.43)	2.40 (0.88)	*F*_(4,627)_ = 208,309^**^, *r^2^* = 0.573	3, 2.5, 2.0 < 1.5, 1.0^*^
EDRL	3.51 (0.70)	2.90 (0.84)	2.55 (0.73)	1.94 (0.72)	1.60 (0.64)	2.46 (0.94)	*F*_(4,627)_ = 103,161^**^, *r^2^*= 0.399	3, 2.5, 2.0 < 1.5, 1.0^*^

SRL, self-regulated learning; NRL, non-regulated learning; DRL, dys-regulated learning; ERL, external-regulated learning; ENRL, external non-regulated learning; EDRL, external dis-regulated learning.

^*^p < 0.01, ^**^p < 0.001.

##### Mediation analysis (Hypothesis 3)

The SPSS v 25-compatible macro created by Hayes (2009, 2013, 2015, 2018, 2022), Hayes and Matthes (2009), Igartua and Hayes (2021), and Hayes and Preacher (2014), Preacher and Hayes (2004), Preacher and Hayes (2008) and Preacher et al. (2007) was used for mediation analysis. Four mediation models were tested, two of which assumed regulatory fatigue as a mediating variable (M) and two of which assumed that behavioral adaptability mediates (M). In all models, combined behavioral regulation was a predictive variable (X). The predicted variable (Y) and the mediating variable (M) was each of the other two as described.

## Results

### Preliminary descriptive results

Descriptive analysis showed the normality of the study variables (see Table 6).

**Table 6:** Normative values for the variables for this sample.

**DV**	**Min**	**Max**	**Mean (SD)**	**ASyM**	**CURTOSIS**	**Kolmogorov–Smirnova**
SR-ER	1.00	−1.67	−0.36 (0.514)	0.033	−0.357	*K*_(624)_ = 0.024, *p < 0.2*00
FATIGUE	1.00	4.25	2.70 (0.523)	−0.091	0.029	*K*_(297)_ = 0.038, *p < 0.2*00
ADAPT	1.17	5.00	3.72 (0.695)	−0.282	0.062	*K*_(329)_ = 0.044, *p* < 0.200
ADAPT-REV	−2.33	1.67	−0.66 (0.886)	−0.061	−0.675	*K*_(282)_ = 0.045, *p < 0.2*00

SR-ER, self-external regulation; FATIGUE, regulatory fatigue; ADAPT, classical adaptability; ADAT-REV, revised adaptability.

### Association and linear prediction (Hypothesis 1)

#### Association and linear prediction between internal-external regulatory factors (SR-ER) and regulatory fatigue (RF)

Bivariate association analysis showed a consistent significant negative association between internal and external regulation and all elements of regulatory fatigue, especially behavioral fatigue. There was also a consistent significant positive association between internal-external non-regulation and regulatory fatigue and between internal-external dysregulation and regulatory fatigue, albeit with a lesser degree of significance (see Table 7).

**Table 7:** Bivariate association relationships between SRL-ERL and regulatory fatigue (*n* = 266).

**SRL-ERL**	**FRCON**	**FRCOG**	**FREMO**	**FRTOT**
SRL	−0.180^**^	−0.410^***^	−0.196^**^	−0.332^***^
NRL	0.276^***^	0.272^***^	0.181^**^	0.317^***^
DRL	0.279^***^	0.024	0.043	0.146^*^
ERL	−0.122^*^	−0.275^***^	−0.174^**^	−0.248^***^
ENRL	0.177^**^	0.166^**^	0.080	0.173^**^
EDRL	0.160^*^	0.022	0.067	0.092

^*^p < 0.05.

^**^p < 0.01.

^***^p < 0.001.

SRL, self-regulated learning; NRL, non-regulated learning; DRL, dys-regulated learning; ERL, external-regulated learning; ENRL, external non-regulated learning; EDRL, external dis-regulated learning; FRCON, behavioral regulatory fatigue; FRCOG, cognitive regulatory fatigue; FREMOC, emotional regulatory fatigue.

Regression analysis showed significant negative prediction by the SR-ER factors and significant positive prediction by NR of total regulatory fatigue [*F*_(6,222)_ = 8,072, *p* < 0.001; *r*^2^ = 0.157). A lack of internal (*B* = −0.236, *p* < 0.001) and external-regulation (*B* = −0.184, *p* < 0.01) together with non-regulatory behavior (*B* = 0.182, *p* < 0.05) significantly predicted total regulatory fatigue. In the case of cognitive regulatory fatigue, the results for prediction were very similar to those for total regulatory fatigue [*F*_(6,2,230)_ = 10,161, *p* < 0.01; *r*^2^ = 0.189). A lack of internal (*B* = −0.284, *p* < 0.001) and external-regulation (*B* = −0.174, *p* < 0.01) together with non-regulatory behavior (*B* = 0.205, *p* < 0.01) significantly predicted cognitive regulatory fatigue. For emotional regulatory fatigue, there was also a significant linear prediction relationship, albeit not as strong [*F*_(6,2,230)_ = 2,862, *p* < 0.01; *r*^2^= 0.045). A lack of internal (*B* = −0.142, *p* < 0.05) and external (*B* = −0.163, *p* < 0.05) were significant predictors of emotional regulatory fatigue, with a greater power of a lack of external regulation (see Table 8).

**Table 8:** Predictive relationships between SRL-ERL and regulatory fatigue (*n* = 266).

**SRL-ERL**	**FRCON**	**FRCOG**	**FREMO**	**FRTOT**
SRL	−0.145^*^	−0.284^***^	−0.142	−0.236^**^
NRL	0.93	0.205^**^	0.094	0.182^*^
DRL	0.218^**^	−0.067	0.006	0.069
ERL	−0.067	−0.174^**^	−0.163^*^	−0.184^**^
ENRL	−0.049	−0.064	−0.093	−0.101
EDRL	0.073	0.071	0.079	0.086

^*^p < 0.05.

^**^p < 0.01.

^***^p < 0.001.

SRL, self-regulated learning; NRL, non-regulated learning; DRL, dys-regulated learning; ERL, external-regulated learning; ENRL, external non-regulated learning; EDRL, external dis-regulated learning; FRCON, behavioral regulatory fatigue; FRCOG, cognitive regulatory fatigue; FREMOC, emotional regulatory fatigue.

#### Association and linear prediction between internal-external regulatory factors (SR-ER) and classical adaptability (ADAPT)

Bivariate association results show a consistent positive association between internal and external regulation and classical adaptation, in all its components. There was also a consistent significant negative association between internal and external non-regulation and adaptability, showing that adaptability requires a high level of self-regulation. In other words, adaptability entails a regulatory effort (see Table 9).

**Table 9:** Bivariate association relationships between SRL-ERL and adaptability (*n* = 266).

**SRL-ERL**	**ADAPCOG**	**ADAPEMO**	**ADAPTOT**
SRL	0.525^***^	0.461^***^	0.537^***^
NRL	−0.157^**^	−0.121^*^	−0.148^**^
DRL	−0.077	0.009	−0.036
ERL	0.267^***^	0.300^***^	0.324^***^
ENRL	−0.116^*^	−0.115^*^	−0.124^*^
EDRL	0.020	0.037	0.042

^*^p < 0.05.

^**^p < 0.01.

^***^p < 0.001.

SRL, self-regulated learning; NRL, non-regulated learning; DRL, dys-regulated learning; ERL, external-regulated learning; ENRL, external non-regulated learning; EDRL, external dis-regulated learning; ADAPCOG, cognitive-behavioral adaptability; ADAPEMO, emotional adaptability; ADAPTOT, total adaptability.

Regression analysis showed significant combined positive prediction for the factors of SR-ER for total classical adaptability [*F*_(6,240)_ = 16,493, *p* < 0.001; *r*^2^ = 0.273), with greater strength for internal regulation (*B* = 0.457, *p* < 0.001) than external regulation (*B* = 0.198, *p* < 0.01). The other non-regulatory and dysregulatory (internal and external) factors did not show significant predictive power. In the case of cognitive adaptability, the results for prediction were also significant [*F*_(6,240)_ = 16,403, *p* < 0.01; *r*^2^ = 0.273). Internal (*B* = 0.471, *p* < 0.001) and external (*B* = 0.112, *p* < 0.05) behavioral regulation positively predicted adaptability whilst dysregulation negatively predicted adaptability (*B* = −0.157, *p* < 0.05). In the case of emotional adaptability, the results for prediction were significant [*F*_(6,247)_ = 11,843, *p* < 0.001; *r*^2^ = 0.204]. Internal (*B* = 0.382, *p* < 0.001) and external (*B* = 0.213, *p* < 0.001) behavioral regulation significantly predicted emotional adaptability (Table 10).

**Table 10:** Predictive relationships between SRL-ERL and behavioral adaptability (*n* = 266).

**SRL-ERL**	**ADAPCOG**	**ADAPEMO**	**ADAPTOT**
SRL	0.504^***^	0.404^***^	0.484^***^
NRL	0.050	0.012	0.033
DRL	−0.149^*^	0.004	−0.071
ERL	0.128^*^	0.215^**^	0.205^**^
ENRL	0.052	0.070	0.069
EDRL	0.010	−0.052	−0.016

^*^p < 0.05.

^**^p < 0.01.

^***^p < 0.001.

SRL, self-regulated learning; NRL, non-regulated learning; DRL, dys-regulated learning; ERL, external-regulated learning; ENRL, external non-regulated learning; EDRL, external dis-regulated learning; ADAPCOG, cognitive-behavioral adaptability; ADAPEMO, emotional adaptability; ADAPTOT, total adaptability.

#### Correlation and linear prediction between internal-external regulation and revised adaptability

The correlation between SR-ER behaviors and reformulated adaptability behaviors showed significant directional correlations, as expected. A significant positive correlation between SR-ER behaviors and adaptability and significant negative correlation with non-adaptability and maladaptability. Also, significant negative correlation between non-regulation and dysregulation with adaptability and significant positive correlation between non-regulation and dysregulation and maladaptability (see Table 11).

**Table 11:** Bivariate association relationships between SRL-ERL and revised adaptability.

**SRL-ERL**	**ADAP**	**NOADAPT**	**DISADAP**
SRL	0.510^***^	−0.358^***^	−0.253^***^
NRL	−0.269^***^	0.440^***^	0.369^***^
DRL	−0.026	0.274^***^	0.326^***^
ERL	0.224^***^	−0.120^*^	−0.204^**^
ENRL	−0.172^**^	0.262^***^	0.338^***^
EDRL	−0.054	0.204^**^	0.241^***^
REGTOT	0.409^***^	−0.707^***^	−0.670^***^

^*^p < 0.05.

^**^p < 0.01.

^***^p < 0.001.

SRL, self-regulated learning; NRL, non-regulated learning; DRL, dys-regulated learning; ERL, external-regulated learning; ENRL, external non-regulated learning; EDRL, external dis-regulated learning; REGTOT, total regulation; ADAPCOG, cognitive-behavioral adaptability; ADAPEMO, emotional adaptability; ADAPTOT, total adaptability.

Predictive results showed significant effects. Thus, there was a significant effect of regulatory factors for adaptation [*F*_(6,232)_ = 14,138, *p* < 0.001; *r*^2^ = 0.269] which was significantly positively predicted by regulatory behavior (*B* = 0.448, *p* < 0.001). Non-adaptation [*F*_(6,232)_ = 14,345, *p* < 0.001; *r*^2^ = 0.252] was significantly negatively predicted by regulatory behavior (*B* = −0.313, *p* < 0.001) and positively predicted by regulatory behavior (*B* = 0.137, *p* < 0.05). Maladaptation [*F*_(6,232)_ = 12,145, *p* < 0.001; *r*^2^ = 0.241] was significantly negatively predicted by regulatory behavior (*B* = −0.147, *p* < 0.001) and positively predicted by dysregulatory behavior (*B* = 0.220, *p* < 0.001).

#### Association and prediction between regulatory fatigue and adaptative behavior

Associations showed were consistently significantly negative between regulatory fatigue and adaptive behavior. It is notable that the strongest association is for cognitive regulatory fatigue (*r* = −0.478; see Table 12).

**Table 12:** Bivariate correlations between regulatory fatigue and adaptability.

**SRL-ERL**	**ADAPCOG**	**ADAPEMO**	**ADAPTOT**
FRCOND	−0.186^**^	−0.234^***^	−0.248^***^
FRCOG	−0.401^***^	−0.458^***^	−0.478^***^
FREMO	−0.280^***^	−0.447^***^	−0.430^***^
FRTOT	−0.373^***^	−0.489^***^	−0.498^***^

^**^p < 0.01.

^***^p < 0.001.

ADAPCOG, cognitive-behavioral adaptability; ADAPEMO, emotional adaptability; ADAPTOT, total adaptability; FRCOND, conductual fatigue; FRCOG, cognitive fatigue; FREMO, emotional fatigue; FRTOT, total fatigue.

Predictive analysis also showed significant effects. Cognitive fatigue (*B* = −0.335, *p* < 0.001) and emotional fatigue (*B* = −0.256*, p* < 0.001) significantly negatively predicted reformulated adaptability [*F*_(3,276)_ = 37,140, *p* < 0.001; *r*^2^ = 0.280]. Cognitive fatigue negatively predicted cognitive adaptability [*B* = −0.343, *p* < 0.001; *F*_(3,282)_ = 19,393, *p* < 0.001; *r*^2^ = 0.166]. Cognitive fatigue (*B* = −0.324, *p* < 0.001) and emotional fatigue (*B* = −0.308, *p* < 0.001) jointly predicted emotional adaptability [*F*_(3,282)_ = 19,393, *p* < 0.001; *r*^2^ = 0.277].

#### Association and prediction between regulatory fatigue and reformulated adaptability

There was a consistent significant negative association between regulatory fatigue and reformulated adaptive behavior. It is notable that the strongest association was between adaptation (*B* = −0.450) and non-adaptation (*B* = −0.442). The revised formulation of adaptation revises the bipolar relationship between regulatory fatigue and adaptation (–) and maladaptation (+) with an intermediate value for non-regulation (+; see Table 13).

**Table 13:** Bivariate correlations between regulatory fatigue and revised adaptability.

**SRL-ERL**	**ADAP**	**NOADAPT**	**DYSADAPT**
FRCOND	−0.195^**^	0.360^***^	0.443^***^
FRCOG	−0.491^***^	0.294^***^	0.265^***^
FREMO	−0.353^***^	0.254^***^	0.331^***^
FRTOT	−0.450^***^	0.390^***^	0.442^***^

^**^p < 0.01.

^***^p < 0.001.

ADAPCOG, cognitive-behavioral adaptability; ADAPEMO, emotional adaptability; ADAPTOT, total adaptability; FRCOND, conductual fatigue; FRCOG, cognitive fatigue; FREMO, emotional fatigue; FRTOT, total fatigue.

Predictive analysis also showed significant effects. Cognitive fatigue (*B* = −0.418, *p* < 0.001) and emotional fatigue (*B* = −0.167*, p* < 0.001) significantly negatively predicted the totally of *reformulated adaptability* [*F*_(3,259)_ = 31,562, *p* < 0.001; *r*^2^ = 0.259]. However, cognitive fatigue (*B* = −0.295, *p* < 0.001) and cognitive fatigue (*B* = −0.208, *p* < 0.001) jointly positively predicted non-adaptability [*F*_(3,262)_ = 17,995, *p* < 0.001; *r*^2^ = 0.161]. Behavioral (*B* = 0.356, *p* < 0.001), cognitive (*B* = 0.114, *p* < 0.05) and emotional (*B* = 0.108, *p* < 0.0*5*) fatigue jointly significantly positively predicted maladaptability [*F*_(3,257)_ = 24,334, *p* < 0.001; *r*^2^ = 0.212], making this level of maladaptation a dysfunctional state of generalized or massive fatigue or ego depletion.

### Inferential results (Hypothesis 2)

#### Effect of the level on the index of combined regulation on regulatory fatigue and adaptability (classical and reformulated) (Hypotheses 2)

There was a significant principal effect of the level of combined regulation on total regulatory fatigue [*F*_(4,224)_ = 7,852, *p* < 0.001, *eta*^2^ = 0.123; power = 0.998]. Error variance showed correct values: *L*_(4,224)_ = 0.313, *p* < 0.869 (mean). There was also a significant principal effect of the level of combined regulation on adaptability [*F*_(4,261)_ = 7,585, *p* < 0.001, *eta*^2^ = 0.104; power = 0.997]. Error variance showed correct values: *L*_(4,312)_ = 0.312, *p* < 0.870 (mean). The significant principal effect of the level of combined regulation on reformulated adaptability was maintained [*F*_(4,226)_ = 17,130, *p* < 0.001, *eta*^2^ = 0.237; power = 0.1.00]. Error variance showed correct values: *L*_(4,221)_ = 0.994, *p* < 0.8439 (media; see Table 14).

**Table 14:** Direct and inferential values (*n* = 229).

**IV**		**DV**	
**Combined regulation**	**Regulatory fatigue**	**Classic adaptability**	**Revised adaptability**
1.00	2.91 (0.42)	3.38 (0.71)	−0.76 (0.62)
1.50	2.86 (0.47)	3.50 (0.81)	−0.56 (0.82)
2.00	2.76 (0.47)	3.61 (0.65)	−0.17 (0.75)
2.50	2.68 (0.51)	3.92 (0.67)	14 (0.84)
3.00	2.37 (0.48)	4.03 (0.66)	65 (0.79)
Total	2.70 (0.51)	3.77 (0.71)	−0.04 (0.89)
*Post-hoc*		3.0 > 2.5, 2.0,1.0^**^	3.0, 2.5 > 2.0 >1.5, 1.0^**^
			2.5 > 2.0, 1.5, 1.0^**^

Combination = level of combined regulation. ^**^p < 0.01.

#### Mediation results (Hypothesis 3)

##### Regulatory fatigue as mediating variable (Hypothesis 3)

The results for the four mediation models tested include various effects worthy of mention. The model with greatest strength and level of significance was Model 2, which showed regulatory fatigue with a strong, significant direct and indirect effect relative to combined regulation and reformulated adaptability. As can be seen in Model 2, combined regulation Reg (COM) has a direct negative effect on regulatory fatigue FRTOT (*M* = −0.75, *p* < 0.01) and a positive direct effect on reformulated adaptability, ADATREV (*X* = 0.48, *p* < 0.001); Regulatory fatigue has a negative effect on reformulated adaptability (*M* = −0.75). It can also be seen that regulatory fatigue in turn has a significant indirect, mediating effect between both (*X* = 0.12, *p* < 0.01; see Table 15).

**Table 15:** Model 4 (Hayes, 2022) of direct and indirect effects (*n* = 193; Bootstrap = 10.000).

**MODEL**	**Variables**	**Effects**	**Significance**	**CI**
Model1.	*Direct effects*			
	Y:TOTADAPT	COM → FRTOT	−0.29^***^	(−0.37, −0.17)
	X:COMB	COM → TOTADAPT	0.18^*^	(0.02, 34)
	M:FRTOT	FR → TOTADAPT	−0.63^***^	(−0.81, −0.45)
	*Combinado (direct and indirect effects)*			
	Y:TOTADAPT	Direct X → Y	0.18^*^	(0.02, 0.34)
	X:DF	Indirect X → Y	0.16^*^	(0.06, 0.20)
	M:FRTOT			
Model2.	*Direct effects*			
	Y:ADAPREV	COMB → FRTOT	−0.25^**^	(−0.37, −0.13)
	X:COMB	COMB → ADAPREV	0.48^***^	(0.30, 0.66)
	M:FRTOT	FR → TOTADAPT	−0.75^***^	(−0.96, −0.54)
	*Combined (direct and indirect effects)*			
	Y:ADAPREV	Direct X → Y	0.48^***^	(0.66, 0.31)
	X:COMB	Indirect X–Y (FRTOT)	0.12^**^	(0.06, 0.19)
	M:FRTOT			
Model3.	*Direct effects*			
	Y:FRTOT	COMB → TOTADAPT	0.34^***^	(0.17, 0.51)
	X:COMB	COMB → FRT	−0.14^**^	(−0.25, −0.03)
	M:TOTADAPT	TOTADAP → FRT	−0.32^***^	(−0.41, −0.23)
	*Combined (direct and indirect effects)*			
	Y:FRTOT	Direct X → Y	−0.14^**^	(0.25, −0.03)
	X:COMB	Indirect X–Y	−0.12^**^	(−0.19, −06)
	M:TOTADAPT			
Model4	*Direct effects*			
	Y:FRTOT	COM → FRTOT	−0.67^**^	(−0.48, 87)
	X:COMB	COMB → ADAPREV	−0.28^**^	(0.35, −0.20)
	M:TOTADAPT	COM → FRTOT	−0.06^***^	(−0.18, −0.05)
	*Combined (direct and indirect effects)*			
	Y:FRTOT	Direct X → Y	−0.067^**^	(0.18, −0.05)
	X:COMB	Indirect X–Y	−0.19^**^	(−0.27, −0.11)
	M:ADAPREV			

COMB, Index of Combined Behavioral Regulation; FRTOT, total regulatory fatigue; TOTADAPT, total adaptability; ADAPREV, reformulated adaptability.

^*^p < 0.05.

^**^p < 0.01.

^***^p < 0.001.

## Discussion

The *objective* of this study was to show the role of regulatory fatigue as a mediating variable in the relationship between combined regulation and behavioral adaptability. The analysis presented is novel for a number of reasons. First, because it incorporates as predictive variable (X) the notion of the *index of combined behavior regulation* (de la Fuente, 2024a,b); combined regulation arises from the *Theory of Self- vs. Externally-Regulated Behavior* (de la Fuente-Arias, 2017; de la Fuente, 2024a,b) which provides a heuristic that can determine an individual's level of regulatory protection or risk in light of their personal characteristics and their environment calculated as an average of possible values falling on one of five levels. Second, because it incorporates as predicted variable (Y) the notion of behavioral adaptability—key to prior theoretical constructs—in a classical (Benlahcene et al., 2024) and reformulated version, which allows determination of the variability of behavior from adaptation to non-adaptation to maladaptation (de la Fuente, 2021a). Finally, because it determines as mediating variable (M) regulatory fatigue as a plausible behavioral mechanism within the framework of SR-ER Theory to explain variability and the shift from a self-regulatory behavior to non-regulation and then to dysregulation. Thus, we have used an experimental concept (regulatory fatigue) for a molar behavioral analysis of university students in an academic setting.

*Hypothesis 1*, that the index of mean regulation (X) would negatively predict regulatory fatigue, is significantly supported by all the results. The association and prediction relationships significantly showed that personal and contextual regulation jointly have a linear effect that tends to lesser regulatory fatigue and greater adaptability. Behavioral non-regulation and dysregulation entailed greater levels of regulatory fatigue. That relationship is of interest because it provides a mechanism whereby dysregulatory or dysfunctional behavior (characterized by behavioral excesses and deficits) on its own entails a component of self-induced fatigue (ego-depletion) by giving rise to maladjustments that lead to the exhaustion of behavioral, cognitive and emotional resources. This insight provides new personal and environmental combined factors that favor regulatory exhaustion of an individual's limited resources, something shown by earlier research (Baumeister and Vohs, 2007; Maranges and Baumeister, 2016; Saleem et al., 2024; Wilson and Talley, 2021).

Combined (internal and external) regulation positively predicted classical adaptability and so supported that hypothesis. Classical adaptability has been defined as a measure of cognitive, behavioral and/or affective adjustment in the face of uncertainty and novel circumstances (Collie and Martin, 2017a,b). Internal and external regulation positively predicted a greater level of adaptability, whilst lower levels of internal and external regulation negatively predicted adaptability. Dysregulatory behavior also negatively predicted the level of classically formulated adaptability. However, there were nuances concerning the type of (cognitive, emotional and behavioral) adaptability; whilst under the classical formulation, cognitive and emotional fatigue negatively predicts adaptability, under the revised formulation all three together predict a state of maladaptability, equivalent to ego-depletion or resource exhaustion. That result helps us to understand how resource exhaustion due to regulatory fatigue predicts a state of dysfunctional maladaptability (Richard-Sephton et al., 2024). Prior research has shown that adaptability significantly positively predicts academic achievement (participation in class, academic enjoyment and positive academic intentions; it negatively predicts self-obstruction and disconnection) and non-academic achievement (self-esteem, life satisfaction, and purpose and goals), effects of sociodemographic factors, prior achievement, assumptions and related correlates (buoyancy and self-regulation) (Martin et al., 2013a,b).

The results for prediction by the components of the index of combined (internal and external) regulation showed an association with and positively predicted adaptability in its revised formulation (de la Fuente, 2021a) and non-regulatory and dysregulatory behavior negatively predicted adaptability in its revised formulation. That corroborates the clear cause and effect relationship between behavioral dysregulation and maladaptation. That result has also been shown previously (Greene et al., 2024).

The negative prediction of adaptability (classical formulation) by regulatory fatigue is also validated by the results. A relationship of association and negative prediction has been shown between classical adaptability and regulatory fatigue and postulated as an interference mechanism (Li, 2021) or a facilitator (Merino-Tejedor et al., 2016; Ran et al., 2023). In similar fashion, relationships with reformulated adaptability had similar directionality, and were even stronger.

*Hypothesis 2*, concerning the inferential discriminatory potential of levels of the index of combined behavioral regulation (regulatory combination) to determine levels of regulatory fatigue and adaptability (classical and reformulated), was strongly and significantly supported. These results provide empirical support for the Index as heuristic with discriminatory value in terms of the gradation it provides of the risk vs. protective factor that it measures. It makes evident the importance of determining and analyzing different regulatory combinations as predicted by Theory of SR-ER (de la Fuente, 2021a; de la Fuente et al., 2022a,b, 2024; de la Fuente and Kauffman, 2025).

*Hypothesis 3* concerning the mediating effect of regulatory fatigue between levels of combined regulation and adaptability (classical and reformulated), is the most important contribution of this work. The explanatory mechanism of the direct and indirect mediating effect of regulatory fatigue between combined regulation and reformulated adaptability has been confirmed. This is the relationship in which mediation was strongest and most significant. That makes it clear that regulatory fatigue is a plausible mechanism for the loss of regulatory capacity, by provoking a shift between states (SR → NR → DR), which ultimately explains levels of adaptive, non-adaptive and maladaptive behavior (AD → NA → DA), as postulated in the reformulation of adaptability (de la Fuente, 2021a) and classical adaptability (Martin et al., 2012). Earlier works have shown the mediating role of regulatory fatigue, consistent with the results of this work, in discussion of ego depletion (Hofmann et al., 2012).

Our findings have a number of implications for the fields of education and health. Thus, the determination of an individual's levels of combined regulation can help to understand which personal and environmental regulatory factors are significant risk vs. protective factors for the individual's mental health (de la Fuente, 2021a; Pachón-Basallo et al., 2022; Rentzios et al., 2025). It should be recalled that a high level of combined regulation means that the individual has a high level of regulation and has an environment that helps them to self-regulate (protective factor), whilst a low level of combined regulation means that the individual has a low or dysregulatory level of self-regulation and has an environment that is dysregulatory (risk factor) (de la Fuente, 2024a,b).

It may also help to predict the degree of regulatory fatigue and burnout which in turn leads to problematic maladjusted or dysfunctional or non-adaptive behaviors seen in post-traumatic stress (Laman-Maharg et al., 2024). It is especially important to understand the dynamic of regulatory change, both in chronic disease (van de Leur et al., 2024) and in learning difficulties (Riccardi, 2024). In both, where a disease or learning difficulty is associated with regulatory fatigue, it makes a lower regulatory threshold more likely, that there will be greater regulatory fatigue and greater loss of the capacity for adaptive behaviors.

This work also has notable *limitations*. First, the nature of the sample. The fact that all participants are university students and aged 18–25 means that the results cannot readily be generalized. Future research should generate results that can be generalized to other sections of the population. Similarly, we cannot assume that our subjects had pathologies sufficient to underpin conclusions concerning transdiagnostic processes. Future research should include studies with clinical and subclinical samples with specific psychopathologies to determine whether the processes put forward work with, for example, in people with ADHD (Burns and Martin, 2014).

## Data Availability

The raw data supporting the conclusions of this article will be made available by the authors, without undue reservation.

## Appendix

**Annex 1:** Utility conceptual model of competence for stress management and psychological wellbeing (de la Fuente and Martínez-Vicente, 2023a).

	**Presage variables**	**Process variables**		**Product variables**
Personal	•Sex/age • Personality • *Internal regulation*	Conceptual	•Learning approaches and styles	•Academic achievement • Academic satisfaction • Academic stress • Flourishing • Physical health • Psychological health
		Procedural	•Coping strategies • Self-regulation • Engagement • Procrastination • Emotional dysregulation • Motivational habits and strategies • Executive function • Burnout	
		Attitudinal	•*Adaptability* • Resilience • Achievement emotions • Action-emotion style • Perfectionism • Character strengths • Assessment anxiety • Academic confidence	
Contextual	•*External regulation* • Family support	•Implementation of teaching process • Teaching-learning experience • Academic stressors		

Intellectual Property Registry ref. n° 00765-01162097 (2022).
