# Supplementary material for: Effect of Combined Regulatory Behavior Index (CRBI) on regulatory fatigue and behavioral adaptability (classical and reformulated) in the university context
Source: Front Psychol. 2025 Aug 4;16:1533725. doi: 10.3389/fpsyg.2025.1533725 (PMC12359180; doi:10.3389/fpsyg.2025.1533725)
Supplement: Supplementary file 1 [file Data_Sheet_1.pdf]

SUPPLEMENTARY MATERIAL

ANEX 1. ADAPTABILITY SCALE –REVISED, AD-NA-DA (de la Fuente, J., 2021; based in: Martin, Nejad, Colmar, & Liem, 2012)

Please read before start.

Please think about how you usually behave in this situation. There are no right or wrong answers. Mark the response that best describes how you behave, where:
1= NOT TRUE OF ME AT ALL
2= ALMOST NEVER TRUE OF ME
3= SELDOM TRUE OF ME
4= SOMEWHAT TRUE OF ME
5= OFTEN TRUE OF ME
6= VERY OFTEN TRUE OF ME
7= ALWAYS TRUE OF ME

Item No.: 1
Statement: I am able to think through a number of possible options to assist me in a new situation.
Options : 1 2 3 4 5 6 7

Item No.: 2
Statement: I am able to revise the way I think about a new situation to help me through it
Options : 1 2 3 4 5 6 7

Item No.: 3
Statement: I am able to adjust my thinking or expectations to assist me in a new situation if necessary
Options : 1 2 3 4 5 6 7

Item No.: 4
Statement: I am able to seek out new information, helpful people, or useful resources to effectively deal with new situations
Options : 1 2 3 4 5 6 7

Item No.: 5
Statement: In uncertain situations, I am able to develop new ways of going about things (e.g. a different way of asking questions or finding information) to help me through
Options : 1 2 3 4 5 6 7

Item No.: 6
Statement: To assist me in a new situation, I am able to change the way I do things if necessary
Options : 1 2 3 4 5 6 7

Item No.: 7
Statement: I am able to reduce negative emotions (e.g. fear) to help me deal with uncertain situations
Options : 1 2 3 4 5 6 7

Item No.: 8
Statement: When uncertainty arises, I am able to minimize frustration or irritation so I can deal with it better
Options : 1 2 3 4 5 6 7

Item No.: 9
Statement: To help me through new situations, I am able to draw on positive feelings and emotions (e.g.: enjoyment, satisfaction)
Options : 1 2 3 4 5 6 7

Item No.: 10

Statement: I do not think through possible options to assist me in a new situation.
Options : 1 2 3 4 5 6 7

Item No.: 11
Statement: I do not usually revise my way of thinking about a new situation to help me get through it.
Options : 1 2 3 4 5 6 7

Item No.: 12
Statement: When facing a new situation in my university work, I do not usually adjust my thinking or expectations, even though it may be necessary.
Options : 1 2 3 4 5 6 7

Item No.: 13
Statement: I do not usually seek out new information, helpful advice, or useful resources to effectively deal with new situations.
Options : 1 2 3 4 5 6 7

Item No.: 14
Statement: In uncertain situations, I do not develop new ways of going about things (e.g., a different way to do something or find information) to help me through.
Options : 1 2 3 4 5 6 7

Item No.: 15
Statement: In new or uncertain situations, I do not usually change the way I do things, even though it may be necessary.
Options : 1 2 3 4 5 6 7

Item No.: 16
Statement: I am not able to minimize negative emotions (e.g., fear) to help me deal with uncertain situations
Options : 1 2 3 4 5 6 7

Item No.: 17
Statement: When a situation of uncertainty arises, I am not able to minimize frustration or irritation in order to deal with it better.
Options : 1 2 3 4 5 6 7

Item No.: 18
Statement: I am not able to draw on positive feelings and emotions (e.g., enjoyment, satisfaction) to help me through new work-related situations.
Options : 1 2 3 4 5 6 7

Item No.: 19
Statement: I think about different options to escape from or to avoid a new, uncertain situation.
Options : 1 2 3 4 5 6 7

Item No.: 20
Statement: I revise my thinking about a new, uncertain situation, in order to avoid it or get out of it.
Options : 1 2 3 4 5 6 7

Item No.: 21
Statement: I can twist my thinking or expectations to deal with a new situation that I do not want.
Options : 1 2 3 4 5 6 7

Item No.: 22
Statement: I seek out new information, advice, or useful resources, even if they are inappropriate or unethical, to effectively deal with new situations.
Options : 1 2 3 4 5 6 7

Item No.: 23

Statement: In uncertain situations, I am able to develop new ways of going about things, even if they are not proper (e.g., different, inappropriate ways to get something done or to get information on how to do an assignment or test).
Options : 1 2 3 4 5 6 7

Item No.: 24
Statement: To assist me in a new situation, I change the way I do things as needed, even if it doesn't seem correct.
Options : 1 2 3 4 5 6 7

Item No.: 25
Statement: I build up my negative emotions (e.g., anger, desperation) to help me deal with uncertain situations.
Options : 1 2 3 4 5 6 7

Item No.: 26
Statement: When a situation with uncertainty arises, I build up my frustration or irritation in order to deal with it better.
Options : 1 2 3 4 5 6 7

Item No.: 27
Statement: I stir up my own negative feelings and emotions (e.g., embarrassment, desperation) to help me get through new, uncertain situations.
Options : 1 2 3 4 5 6 7
